# Drug resistance‐related microRNAs in osteosarcoma: Translating basic evidence into therapeutic strategies

**DOI:** 10.1111/jcmm.14064

**Published:** 2019-02-05

**Authors:** Ruiling Chen, Gangyang Wang, Ying Zheng, Yingqi Hua, Zhengdong Cai

**Affiliations:** ^1^ Department of Orthopedics Shanghai Bone Tumor Institute, Shanghai General Hospital, Shanghai Jiao Tong University School of Medicine Shanghai China

**Keywords:** biomarker, drug resistance, miRNA, osteosarcoma, therapeutic target

## Abstract

Although the application of multiple chemotherapy brought revolutionary changes to improve overall survival of osteosarcoma patients, the existence of multidrug resistance (MDR) has become a great challenge for successful osteosarcoma treatment in recent decades. Substantial studies have revealed various underlying mechanisms of MDR in cancers. As for osteosarcoma, evidence has highlighted that microRNAs (miRNAs) can mediate in the processes of DNA damage response, apoptosis avoidance, autophagy induction, activation of cancer stem cells, and signal transduction. Besides, these drug resistance‐related miRNAs showed much promise for serving as candidates for predictive biomarkers of poor outcomes and shorter survival time, and therapeutic targets to reverse drug resistance and overcome treatment refractoriness. This review aims to demonstrate the potential molecular mechanisms of miRNAs‐regulated drug resistance in osteosarcoma, and provide insight in translating basic evidence into therapeutic strategies.

## 1 INTRODUCTION

Osteosarcoma (OS) with great tumour malignancy, has a predilection for children and adolescents, principally emerging in the metaphysis of long bones.^1^ The peak age of OS occurrence is approximately 16 years, which was substantiated to have a close association with skeletal growth rate.^2^ Because of its strong tendency to extensive metastasis and tumour relapse, OS consequently causes high mortality and poses a great threat of life to children and adolescents. With the emergence of next‐generation sequencing, OS was gradually discovered to have a rather complicated genetic background.^3^ The inactivation of tumour suppressor genes *TP53* and/or *RB1* was corroborated to remarkably induce OS tumourigenesis.^4^ The congenital mutations of *TP53* and/or *RB1* are enough for developing tumour, but the occurrence rate of these congenital mutations was underestimated before.^5^ Currently, the combination of surgical resection and multiple chemotherapy including neoadjuvant therapy, has been standardized for OS clinical remedy since 1970s. This regimen tremendously ameliorated symptoms and extended overall survival time of OS patients.^6^ However, there exists a low response to therapeutic drugs in many OS patients, which is responsible for their subsequent aggressive progression and unfavourable outcomes. It is noted that the 5‐year survival rate has remained at the level of 65%‐75% in recent three decades, even with substantial research progress in OS clinical treatment approaches.^7^, ^8^, ^9^ Obviously, distant metastasis, tumour recurrence and drug resistance are three pivotal reasons for the treatment refractoriness of OS. Much attention should be paid to better decipher and understand the underlying molecular mechanisms. Hence, it would be conceivable that molecules implicated in these mechanisms can serve as therapeutic targets for extending survival time of OS patients. Noticeably, extensive evidence has supported the involvement of miRNAs in OS pathogenesis.

In recent years, miRNAs have been explored to have a close connection to the mechanisms of pathogenesis and drug resistance in different cancer types, and establish a competitive endogenous RNA regulatory network that remains to be investigated.^10^, ^11^, ^12^, ^13^ Inspiringly, miRNAs seem to play an emerging role in OS drug resistance.^14^, ^15^ This might provide a brand‐new insight in seeking for promising prognostic biomarkers and therapeutic targets for successful treatment in OS. To our knowledge, a single miRNA can target at least 200 genes involved in one signalling pathway or diverse signalling pathways.^16^ Therefore, miRNAs might be valuable and effective for treating cancers with inherent heterogeneity and abnormality of multiple genes, among which OS can be taken as a good example.

In this review, we will elaborate on the emerging role of miRNAs in OS drug resistance under the mechanisms of DNA damage response, apoptosis avoidance, autophagy induction, activation of cancer stem cells (CSCs), and alteration in signal pathways. Also, we will provide insight in the potential clinical utility of these miRNAs as promising biomarkers and therapeutic targets to reverse chemoresistance.

## 2 BIOGENESIS AND BIOLOGICAL FUNCTION OF miRNAs

**Figure 1:**
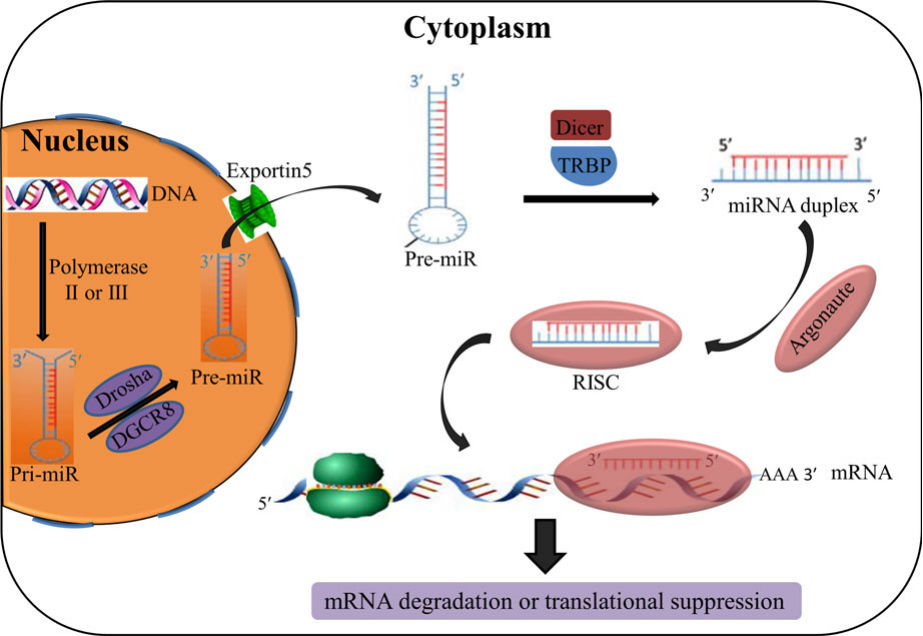
Biogenesis and biological function of miRNAs. First, a specific miRNA gene transcribes into pri‐miRNA through polymerase II or III in the nucleus. Next, Drosha cleaved the hairpin structure of pri‐miRNA to produce pre‐miRNA which is subsequently exported to the cytoplasm by Exportin5. Then, the miRNA duplex is released after the excision of Dicer. After that, a combination of miRNA duplex and Argonaute protein forms a RNA‐induced silencing complex (RISC), in which the passenger strand of miRNA is degraded. Finally, RISC causes mRNA degradation or translational suppression by targeting the 3′‐UTR of mRNA

microRNAs (miRNAs) were first discovered by Victor Ambros et  al in 1993, and perceived as endogenous small RNA molecules with biologically regulatory functions.^17^ They are broadly conserved sequences among species with only 18‐25 nucleotides in length for their mature forms, and have regulatory roles in gene expression at the post‐transcriptional level.^18^, ^19^ Through binding to the 3′‐untranslated region (3′‐UTR) perfectly or imperfectly, they consequently contribute to the translational suppression or the degradation of diverse target mRNAs.^20^ The detailed biogenesis process and functional mechanism of miRNAs have been well‐elucidated (Figure 1). It has been estimated that over 70% of human genome DNA has transcripts. Among them, about 2% transcripts code for protein synthesis and 3% can transcribe endogenous miRNAs. Of note, over 30% of human genes are under the regulation of miRNAs.^19^, ^21^ Intriguingly, a single small miRNA can interact with several regions of one or multiple target mRNAs. Conversely, a mRNA can be modulated by a multitude of miRNAs simultaneously, which is a unique advantage of miRNAs for cancer treatment.

Evidence has accumulated that miRNAs participate in various biological processes,^22^, ^23^ such as development, proliferation, differentiation, apoptosis, cell cycle, and metabolism, together with some human diseases^24^ including cancer.^25^ These deregulated miRNAs can be categorized as oncogenic ones and tumour suppressor ones. They play a regulatory function in tumourigenesis, progression, or chemosensitivity of different cancers. Besides, some miRNAs were reported to possess clinical values as predictive factors or therapeutic targets.^26^, ^27^, ^28^ Noticeably, the emerging role of miRNAs in OS chemoresistance has been reported in recent studies, holding promise for improving the quality of life in OS patients.^15^

## 3 miRNAs‐MODULATED DRUG RESISTANCE IN CANCER

Although the application of chemotherapeutic agents contributes to effective cancer treatment to a large extent, the occurrence of acquired multidrug resistance (MDR) remains a tough issue that ought to be solved. Substantial studies have discovered several universal mechanisms underlying acquired MDR,^29^ including drug transport, drug metabolism, aberrant drug targets, DNA damage response, apoptosis evasion, autophagy, epithelial‐to‐mesenchymal transition (EMT), and activation of CSCs.

Drug transport mechanism has been well‐studied in cancer MDR, which is closely associated with up‐regulated drug transport proteins presenting on the surface of cytoplasmic membrane, that is, ATP‐binding cassette (ABC) transporters.^30^ Drug metabolism is a complicated process of xenobiotics detoxification with the participation of drug metabolism enzymes (DMEs) and the consequent metabolites are transported by ABC transporters.^31^ As we can see, the concerted efforts of DMEs and ABC transporters finally lead to the decreased drug accumulation in the cytoplasm to reduce drug toxicity. DNA damage response (DDR) is a cellular stress response to DNA damage caused by cytotoxic drugs endogenously or exogenously. It aims to repair existing DNA lesions by arresting cell cycle temporarily, and prevent further or irretrievable damage such as cell senescence and apoptosis.^32^ Therefore, the enhanced DNA repair can promote cell viability and resistance to cytotoxicity. Programmed cell death, an integrated concept of apoptosis, autophagy, and programmed necrosis, is an intracellular program triggered in the context of adverse conditions to determine the ultimate fate of cells, namely, survival or death. Interestingly, in malignant cells, apoptosis and programmed necrosis are invariably associated with death, while autophagy executes a dual role.^33^ Furthermore, mechanisms modulated by apoptosis or autophagy have been confirmed to contribute to enhanced drug resistance.

Recently, ever‐growing evidence has shown that exosomes and miRNAs can also play a significant role in drug responsiveness of cancers including OS.^15^, ^34^ Extracellular tumour‐derived exosomes can transfer MDR‐related miRNAs through 40‐150 nm vesicles to recipient cells. Of note, miRNAs can modulate all of the above mechanisms of MDR because of their extensive regulation in gene expression in various cancers.^35^, ^36^, ^37^, ^38^ Therefore, miRNA can be viewed as a pivotal mediator of cancer chemoresistance. In spite of these miRNAs‐modulated drug resistance mechanisms, another challenge we faced is to identify useful targets that can effectively overcome MDR. Given that chemotherapy insensitivity is usually blamed for the rapid growth of local tumours and widespread metastasis to distant organs, it is still an urgent duty to have a thorough understanding of MDR modulated by miRNAs and deeply explore viable methods to reverse drug resistance.

## 4 THE ROLE OF miRNAs IN OSTEOSARCOMA DRUG RESISTANCE

**Figure 2:**
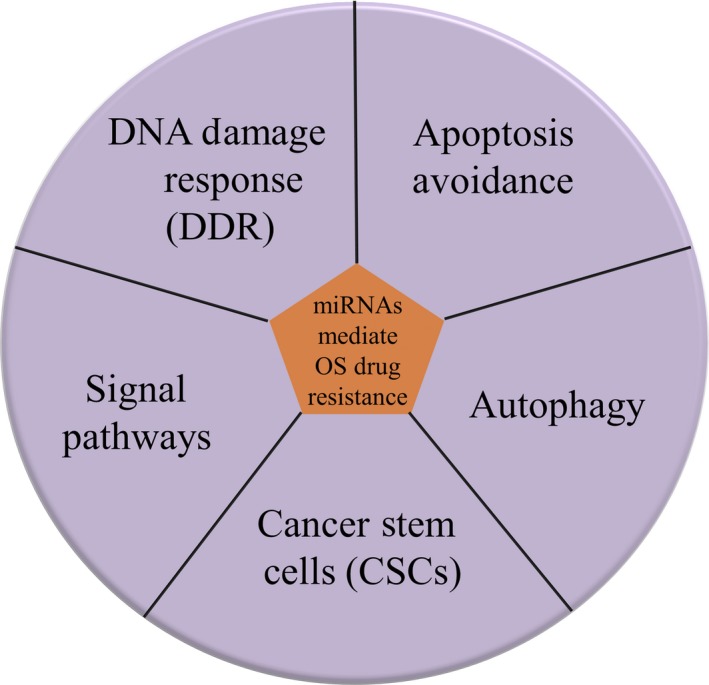
MiRNAs modulate OS drug resistance through several mechanisms

Clinically, the traditional first‐line chemotherapy regimen for OS patients is a combination of doxorubicin (DOX), cisplatin (CDDP), and methotrexate (MTX). The following resistance to these anticancer drugs is a common phenomenon and contributes to poor clinical outcomes. The underlying mechanisms now have been unveiled.^39^ With deep investigations of miRNAs in recent years, numerous studies have validated the involvement of miRNAs in OS drug resistance, in addition to tumour initiation and progression.^40^ These oncogenic or tumour suppressor miRNAs role in chemotherapy sensitivity by the mechanisms of DDR, apoptosis avoidance, autophagy induction, activation of CSCs, and alteration in signal pathways (Figure 2). Besides, they show much promise for predicting clinical outcomes in clinical practice.

### 4.1 DNA damage response

Cytotoxic agents can cause cellular DNA damage and initiate a cellular stress response called DDR, which aims to repair existing DNA lesions through temporary cell cycle arrest and protect cells from irreversible damage.^32^ To our knowledge, the DDR process contains DNA tolerance mechanisms, base excision repair, nucleotide excision repair, mismatch repair, and DNA double‐strand break repair.^41^ It has been reported that there exists an interplay between DDR genes and noncoding RNAs (ncRNAs) including miRNAs in cancer.^42^, ^43^ Several recent studies have shown that miRNAs can be a regulator of OS drug resistance via involving in DDR mechanism (Table 1).

**Table 1:** MiRNAs regulate DDR, autophagy, CSCs, and signal pathways

Mechanism	microRNA	Alteration	Target gene	OS‐derived cell line	Resistant to	References
DNA damage response	miR‐124	↓	ATMIN; PARP1	U2OS	CPT, VP‐16 and DOX	46
	miR‐15b	↓	WEE1	KHOS, KHOSmr, U2OS, and U2OSmr	DOX	52
Autophagy	miR‐101	↓	Not defined	U2OS	DOX	86
	miR‐22	↓	HMGB1	U2OS and MG63	DOX and CDDP	87, 88
	miR‐30a	↓	Beclin‐1	MG63/Dox resistant cell line	DOX	91
	miR‐199a‐5p	↓	Beclin‐1	MG63	CDDP	92
	miR‐155	↑	Not defined	Saos2 and MG63	DOX and CDDP	94
	miR‐140‐5p	↑	IP3K2	Saos2 and MG63	DOX and CDDP	95
Cancer stem cells	miR‐143	↓	Not defined	U2OS and Saos2	DOX	108
	miR‐let‐7	↓	Not defined	KPD, U2OS and Saos2	Not defined	110
	miR‐let‐7d	↓or↑	Multiple genes	3AB‐OS CSC line	Not defined	111
	miR‐29b‐1	↓	Multiple genes	3AB‐OS CSC line	DOX, CDDP and VP‐16	112
Signal pathways	miR‐34c	↓	Notch1; LEF1	U2OS and MG63	DOX, CDDP and MTX	115
	miR‐34b	↓	PAK1; ABCB1	MG63/ADM resistant cell line	DOX, GEM and MTX	116
	miR‐497	↓	VEGFA	Saos2	CDDP	117
	miR‐221	↑	PTEN	SOSP‐9607 and MG63	CDDP	118
	miR‐146b‐5p	↑	ZNRF3	U2OS and MG63	DOX, CDDP and MTX	119

ATMIN, ataxia telangiectasia mutated interactor; PARP1, poly (ADP‐ribose) polymerase 1; HMGB1, high‐mobility group box 1; IP3K2, inositol 1,4,5‐trisphosphate kinase 2; LEF1, lymphoid enhancer‐binding factor 1; PAK1, p21‐activated protein kinase 1; ABCB1, ATP‐binding cassette, subfamily B, member 1; VEGFA, vascular endothelial growth factor A; PTEN, phosphatase and tensin homolog; ZNRF3, zinc and ring finger 3; CPT, camptothecin; VP‐16, etoposide; DOX, doxorubicin; CDDP, cisplatin; MTX, methotrexate; GEM, gemcitabine (↑upregulation, ↓downregulation).

MiR‐124 was previously reported to regulate glucocorticoid resistance in haematological malignancies, for which glucocorticoid is common therapeutic drug.^44^, ^45^ Up‐regulated miR‐124 was newly shown to enhance cell response to diverse DNA‐damaging drugs by binding to the 3′‐UTR of *ATMIN* and *PARP1* mRNAs in U2OS cells.^46^ Protein PARP1, an abbreviation of poly (ADP‐ribose) polymerase 1, is well‐known to attract DNA repair proteins for repair through binding to DNA breaks.^47^ Its inhibitors have been validated to sensitize cancer cells and have an anticancer effect in various cancers.^48^, ^49^ Protein ATMIN (ATM interactor) interacts with a significant DNA damage checkpoint kinase, ataxia telangiectasia mutated (ATM), and regulates ATM activity for DNA repair.^50^ The role of miR‐15b in cancer drug resistance has been reported in the last decade.^51^ A recent study^52^ first pointed out a significant decrease in miR‐15b in OS MDR cell lines and identified *WEE1* mRNA as its direct target. *WEE1* gene codes for a protein kinase to modulate the G2 checkpoint in response to DNA damage. Besides, a restoration of miR‐15b was observed to suppress *WEE1* and partially reverse drug resistance in vitro. By establishing a MDR models of OS, Zhenfeng Duan et al discovered an attenuate resistance to DOX after systemic administration of miR‐15b mimics.

As we all know, intracellular genomic instability is an intrinsic hallmark of tumourigenesis and tumour progression. Some cancer cells rely on a limited set of repair mechanisms for survival. Studies have found that disruption of DNA damage repair pathways can be utilized for current anticancer therapies.^53^, ^54^ However, it is still obscure in OS chemotherapy and requires deeper exploration of potential mechanisms of miRNAs‐regulated DDR in OS.

### 4.2 Apoptosis avoidance

**Figure 3:**
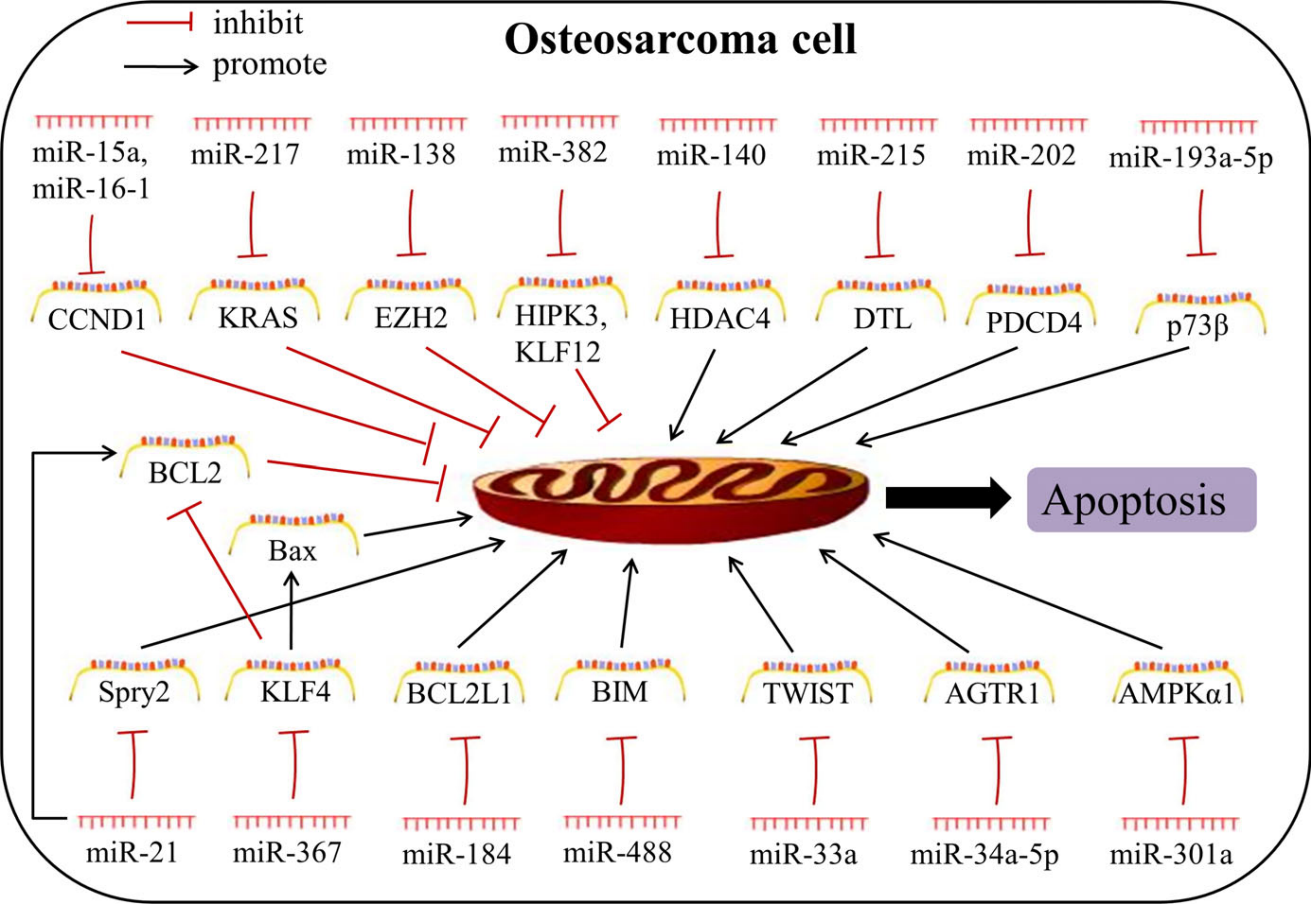
Aberrant expression of apoptosis‐related miRNAs

Cell apoptosis, characterized by permanent cell cycle arrest, is a complicated prodeath process elicited by activation of a cascade of intracellular caspases.^55^, ^56^ It is believed to predict the treatment effect of anticancer drugs. The perturbations in apoptotic process result in uncontrolled cell proliferation, which is an outward manifestation of resistant cancer cells. Previous studies suggest that molecules implicated in apoptotic process can serve as effective targets to reverse cancer drug resistance.^57^ Inspiringly, recent researches have demonstrated that miRNAs regulate cell apoptosis by affecting apoptosis‐related proteins to obviously influence chemotherapy sensitivity of OS cells (Figure 3, Table 2).

**Table 2:** MiRNAs involved in apoptosis avoidance

microRNA	Alteration	Target gene	OS‐derived cell lines	Resistant to	References
miR‐126	↓	Not defined	U2OS	EGCG	58
miR‐15a, miR‐16‐1	↓	CCND1	SOSP‐9607	Not defined	59
miR‐217	↓	KRAS	143B	CDDP	60
miR‐138	↓	EZH2	HOS, Saos‐2, MG63, U2OS	CDDP	61
miR‐382	↓	HIPK3; KLF12	MNNG/HOS, U2OS and MG63	DOX, CDDP and MTX	62
miR‐140	↑	HDAC4	U2OS	MTX and 5‐FU	14
miR‐215	↑	DTL	U2OS and MG63	MTX and tomudex	63
miR‐301a	↑	AMPKα1	U2OS and MG63	DOX	64
miR‐21	↑	Not defined	MG63	CDDP	66
miR‐21	↑	Spry2	U2OS	CDDP	67
miR‐184	↑	BCL2L1	U2OS and MG63	DOX	68
miR‐367	↑	KLF4	MG63, U2OS and Saos2	DOX	69
miR‐488	↑	Bim	MG63 Saos2 and G293	DOX	70
miR‐202	↑	PDCD4	U2OS and G292	DOX	71
miR‐33a	↑	TWIST	Saos2 and MG63	CDDP	72
miR‐193a‐5p	↑	TAp73β	143B, MNNG/HOS, Saos2, SJSA1, MG63, U2OS and CAL‐72	CDDP	73
miR‐34a‐5p	↑	AGTR1	SJSA1 and G292	CDDP, VP‐16, CDDP and CBP	75

CCND1, Cyclin D1; EZH2, enhancer of zeste 2 polycomb repressive complex 2 subunit; HIPK3, homeodomain interacting protein kinase 3; KLF12, Kruppel‐like factor 12; HDAC4, Histone deacetylase 4; DTL, denticleless protein homolog; Spry2, Sprouty homolog 2; BCL2L1, Bcl‐2‐like protein 1; KLF4, Kruppel‐like factor 4; Bim, Bcl‐2‐interacting mediator of cell death; PDCD4, programmed cell death 4; AGTR1,angiotensin II type 1 receptor; Epigallocatechin‐3‐gallate (EGCG), doxorubicin (DOX), cisplatin (CDDP), methotrexate (MTX), 5‐fluorouracil (5‐FU), etoposide (VP‐16), carboplatin (CBP) (↑upregulation, ↓downregulation).

MiR‐126 is a key regulator in inflammation and angiogenesis. The low expression level of miR‐126 has been commonly reported in cancers. Up‐regulated miR‐126 promoted cell sensitivity to Epigallocatechin‐3‐gallate (EGCG) by enhancing cell apoptosis in U2OS cells.^58^ Overexpressed miR‐15a and miR‐16‐1 induced apoptosis and cell cycle arrest in SOSP‐9607 cell line and post‐transcriptionally modulated cyclin d1 (*CCND1*) expression via directly targeting the 3′‐UTR of *CCND1*.^59^ CCND1 is a key regulator in the G1 phase, a pivotal cell cycle phase in response to extracellular cues, and is usually up‐regulated in multiple cancers. After adding quercetin drug, there showed enhanced sensitivity to CDDP, the up‐regulation of miR‐217, and down‐regulation of its target *KRAS* at the level of mRNAs and proteins. This implied that quercetin increased CDDP‐induced cytotoxicity through the miR‐217‐KRAS axis.^60^ Reduced expression of miR‐138 was assessed in OS tissues and cell lines, and miR‐138 transfection suppressed cell proliferation, induced cell apoptosis, and increased drug responsiveness by binding to *EZH2*.^61^ MiR‐382 was detected to decrease in OS specimens with chemoresistance compared to those with chemosensitivity. Further study showed that elevated miR‐382 inhibited cell growth and drug resistance via interacting with *KLF12* and *HIPK3*, respectively. Besides, Meng Xu et al confirmed a relationship between miR‐382 and genes *KLF12* and *HIPK3* by using a MNNG/HOS xenograft model.^62^

Expression of miR‐140 is ubiquitous in chondrocyte for bone development during embryonic period. The oncogenic role of miR‐140 in drug resistance relied on the existence of functional wild‐type p53, for which this study was performed in U2OS cells. MiR‐140 inhibited the level of histone deacetylase 4 (*HDAC4*) and contributed to chemoresistance through G1 and G2 phase arrest and p21 up‐regulation.^14^ Amplified miR‐215 inhibited cell proliferation through G2 phase arrest and promoted chemotherapy insensitivity to MTX and TDX, accompanied by overexpression of p21 in a p53‐dependent manner.^63^ Elevated miR‐301a enhanced drug resistance because of apoptosis avoidance by directly targeting *AMPKa1*.^64^ It has been identified that miR‐21 mostly exerts oncogenic roles in cancers including OS.^65^ A study revealed that Bcl‐2 expression had a positive connection with miR‐21 which inhibited apoptosis and induced a resistance to CDDP, while Bcl‐2 siRNA ameliorated miR‐21‐induced resistance.^66^ Another recent study identified *Spry2* as a direct target of miR‐21, and confirmed the positive role of miR‐21in OS drug resistance.^67^ Time‐dependent expression of miR‐184 was observed in OS cells treated with DOX and up‐regulated miR‐184 caused a poor drug response through targeting bcl‐2‐like protein 1 (*BCL2L1*).^68^ MiR‐367 negatively modulated DOX‐induced apoptosis via coupling with *KLF4*, which could enhance cell apoptosis by regulating Bax and Bcl‐2.^69^ MiR‐488 was induced by hypoxia because HIF1‐α could interact with the hypoxia response element (HRE) within miR‐488 promoter. Overexpressed miR‐488 resulted in apoptosis avoidance, drug resistance, and promoted proliferation by binding to bcl‐2‐interacting mediator (*BIM*) of cell death, while an opposite result was obtained via using miR‐488 inhibitor.^70^ MiR‐202 was found to be up‐regulated in OS tissues and could be induced by TGF‐β1 in OS cells. MiR‐202 mimics transfection led to a significant promotion of chemoresistance together with a decrease in the expression of an apoptosis‐related protein PDCD4, while miR‐202 inhibitor triggered an opposite effect.^71^ Increased miR‐33a was observed in chemo‐resistant OS and in vitro data showed that miR‐33a enhanced drug resistance by inhibiting CDDP‐induced apoptosis in OS cells with a negative regulation of *TWIST*. On the contrary, decreased miR‐33a by antagomir‐33a promoted cell apoptosis and increased levels of *TWIST* mRNA.^72^ Oncogenic miRNA‐193a‐5p modulated cell viability, colony‐forming capacity, and CDDP‐induced apoptosis in OS cells through targeting *TAp73β*,^73^ an isoform of P73 which belongs to the P53‐related transcription factor family and regulates genome stability and chemosensitivity.^74^ MiR‐34a‐5p was discovered to promote MDR of OS by targeting angiotensin II type 1 receptor (*AGTR1*) in sensitive (G292) and resistant (SJSA1) OS cells, and function of miR‐34a‐5p in drug resistance was further verified in G292 and SJSA1‐derived xenografts.^75^

Collectively, these oncogenic or tumour suppressor miRNAs contribute to OS drug resistance by regulating expression of apoptosis‐related genes to avoid cell apoptosis, such as *CCND1* and *BCL2*. Considering that BCL2 is a classic anti‐apoptotic protein that promotes cell survival by inhibiting activation of a caspase cascade, and is associated with several miRNAs in OS chemoresistance, it's presumable that BCL2 might be critical for the reversal of MDR in OS. However, further identification and confirmation of the above miRNAs is needed and great efforts should be invested to translate these findings into clinical applications.

### 4.3 Autophagy induction

On one hand, autophagy refers to a lysosomal degradation pathway by secluding damaged or excess cellular molecules and organelles within autophagosomes and clearing them to keep cellular homeostasis.^76^ On the other hand, it's a protective prosurvival pathway by sustaining a balance among the synthesis, degradation, and succeeding recycling of essential molecules in the condition of nutrient deprivation.^77^ Conversely, autophagy will trigger cell death in the context of excessive loss of proteins, indicating that autophagy can exert paradoxical roles.^78^ Accumulated evidence has highlighted the participation of autophagy regulation in cancer diseases including OS,^79^, ^80^, ^81^ and revealed the promoted activity of this degradative pathway after administration of cytotoxic drugs to acquire drug resistance.^82^, ^83^, ^84^ Recently, the involvement of autophagy modulated by miRNAs in OS drug resistance has been explored (Table 1).

MiR‐101 is viewed as an important regulator in fibrotic diseases and is used as therapeutic agents.^85^ But except for that, it is also newly reported in cancer drug resistance. MiR‐101 significantly blocked the expression of autophagy‐related gene in U2OS cells and promoted cell sensitivity to DOX treatment.^86^ MiR‐22 was reported to couple with high‐mobility group box 1 (*HMGB1*) and suppress HMGB1‐modulated autophagy in OS cells treated with DOX and CDDP.^87^, ^88^ Previous studies imply that HMGB1, a chromatin‐binding nuclear protein, can regulate the balance of autophagy and apoptosis, and promote drug resistance by facilitating autophagy in OS cells with administration of agents.^89^, ^90^ It was confirmed that miR‐30a targeting *Beclin‐1* reduced chemoresistance to DOX via inhibition of Beclin‐1‐regulated autophagy in vitro.^91^ MiR‐199a‐5p also bound to Beclin‐1 contributing to blockage of autophagy and CDDP‐induced cytotoxicity in MG63 cells.^92^ Multifunctional miR‐155 is enriched and important in cellular immune system, and its overexpression is well‐known to result in cancer development and drug resistance. The miR‐155‐based therapy has been commonly considered in cancer treatment.^93^ A recent study revealed that elevated expression of miR‐155 promoted autophagy induced by anti‐cancer drugs and increased cell viability to modulate drug resistance in OS cells.^94^ MiR‐140‐5p played a positive role in OS drug resistance through induction of autophagy with a direct interaction with inositol 1,4,5‐trisphosphate kinase 2 (*IP3k2*).^95^ Since autophagy is a double‐edged sword in the process of biological degradation, and tight control of autophagy is beneficial for the survival of normal or cancer cells, it would be a considerable notion that manipulation of autophagy can be applied in cancer therapy by inhibiting its protective function and inducing cell death instead.^96^, ^97^ This has been studied preclinically in its infancy in OS treatment.^98^, ^99^ Therefore, it demands for further investigations in OS to seek for effective therapeutic drugs targeting miRNAs‐modulated autophagy.

### 4.4 Activation of cancer stem cells

Cancer stem cells (CSCs) refer to a small subpopulation of cells possessing competences of self‐renewal and differentiation, holding malignant potential, showing resistance to therapeutic drugs by expressing ABC transporters, and serving as the source of metastatic and recurrent tumours. Hence, it is universally perceived that an eradication of CSCs is pivotal but challenging for the successful treatment of cancers.^100^, ^101^ Ever‐growing evidence indicates that therapeutic approaches targeting CSCs can effectively halt tumour development and ameliorate patient prognosis, which has also been reported in OS.^102^, ^103^, ^104^ Recently, ncRNAs including miRNAs and lncRNAs have been reported to participate in the maintenance of the CSC phenotype, which brought great benefits to better understand CSCs by further exploring CSCs‐related ncRNAs. For example, hypoxia‐inducible factor‐2α promoter upstream transcript (HIF2PUT) was the first lncRNA reported to play a role in OS‐CSCs with expression of CD133.^105^ Remarkably, several current studies shed light on the involvement of miRNAs in OS‐derived CSCs, which needs much more investigations to have a good understanding of potential mechanisms for their future applications in OS treatment (Table 1).

MiR‐143 is viewed as a novel regulator in type II diabetes, which can specially suppresses insulin‐AKT pathway and causes insulin resistance.^106^ Besides, chemically modified miR‐143 has been considered as a RNA medicine for treating colorectal tumours.^107^ A study reported a reduced level of miR‐143 in OS patients with drug treatments, which contributed to enhanced chemoresistance by apoptosis avoidance and activation of autophagy and ALDH1^+^CD133^+^ cells.^108^ It is acknowledged that ALDH1 and CD133 are common cancer stem cell markers for identifying and selecting CSCs.^109^ Eva Wessel Stratford et al demonstrated that a specific inhibitor of tankyrase JW74 could delay cell cycle progression, induce apoptosis and osteogenic differentiation in OS cells, and up‐regulate miRNA let‐7. MiRNA let‐7 is a main regulator of differentiation and associated with CSC phenotype.^110^ Notably, the increased level of miRNA let‐7 induced by JW74 triggered poorly differentiated cancer cells to differentiate, implying that tankyrase can modulate a switch between stemness and differentiation through dysregulated miRNAs. Subsequently, a recent study unveiled both tumour suppressor and oncogenic roles of miR‐let‐7d, a member of let‐7 family. MiR‐let‐7d can modulate multiple associated genes in 3AB‐OS cells which is a CSC line derived from MG63 cells.^111^ A significant decrease in miR‐29b‐1 was detected in 3AB‐OS cells, and miR‐29b‐1 was unveiled to negatively regulate stem cell markers including Oct3/4, Sox2, Nanog, CD133 and N‐Myc, cell cycle‐related markers such as CCND2, E2F1, and E2F2, and anti‐apoptotic markers like Bcl‐2 and IAP‐2. Therefore, elevated miR‐29b‐1 suppressed stemness properties, cell proliferation, self‐renewal, and drug resistance of 3AB‐OS CSCs via direct or indirect interaction with these mRNAs.^112^ These study findings reveal an internal connection between miRNAs and CSCs in OS, providing a new perspective for the study of CSCs to improve prognosis of OS patients.

### 4.5 Alteration in signal pathways

Abnormal signal transduction pathways seem to regulate initiation, progression, and chemotherapy sensitivity to anticancer drugs in various cancers. There are several common OS‐associated signal pathways which include Wnt/β‐catenin, PI3K/Akt, IGFIR, Notch, TGF‐β, and so on. Wnt/β‐catenin pathway plays a role in osteoblast differentiation and was reported to be the most important one for OS tumourigenesis.^113^ PI3K/Akt pathway is another crucial pathway participating in OS pathogenesis, and has been recently confirmed as a key vulnerability for OS treatment.^114^ Some recent studies have demonstrated that miRNAs could elicit aberrant activities of OS‐associated pathways to affect chemosensitivity (Table 1).

Decreased miR‐34c resulted in OS metastasis and chemoresistance by directly targeting the 3′‐UTR of *Notch1* and *LEF1*.^115^ Sirolimus was reported to induce cell apoptosis and increase cell sensitivity to therapeutic drugs with an up‐regulation of miR‐34b targeting p21‐activated protein kinase 1 (*PAK1*) and *ABCB1*.^116^ The expression level of miR‐497 was reduced in OS tissues, contributing to enhanced activation of PI3K/Akt signalling and resistance to CDDP through binding to vascular endothelial growth factor A (*VEGFA*). Further functional confirmation was executed in Saos2 xenograft tumour model.^117^ MiR‐221 was overexpressed in OS samples. It repressed cell apoptosis, promoted cell survival, and increased CDDP resistance due to its direct interaction with *PTEN*, which causes the activation of PI3K/Akt pathway.^118^ Inactivation of PI3K/Akt pathway has been revealed to augment expression of Bcl‐2, CCND1, both of which were under the regulation of miR‐221. In OS tissues treated with anticancer drugs, up‐regulated miR‐146b‐5p was observed to facilitate proliferation, migration, and metastasis by positively regulating *MMP‐16*, and resistance to chemotherapy via negatively regulating zinc and ring finger 3 (*ZNRF3*), a molecule inactivating Wnt/β‐catenin signalling pathway.^119^ Generally, these results provide an appealing strategy to target miRNAs implicated in signal pathways to improve OS therapeutic effectiveness.

## 5 THE CLINICAL UTILITY OF miRNAs IN OSTEOSARCOMA DRUG RESISTANCE

According to the above preclinical studies, these drug resistance‐related miRNAs are expected to supplement or replace existing biomarkers of diagnosis or prognosis, and serve as promising candidates for therapeutic targets to overcome drug resistance in the coming future.^40^

Several drug resistance‐related miRNAs were mentioned to have a predictive role in clinical prognosis and survival time of OS patients. Clinically, reduced miR‐382 was correlated with unfavourable prognosis in OS patients, due to its potent effect on chemoresistance to anticancer drugs.^62^ OS patients with low expression level of miR‐15b had obviously poor prognosis and shorter survival times because of chemotherapy resistance.^52^ A low expression level of miR‐143 was observed in OS samples, which had a significant connection with poor outcomes and shorter survival of OS patients with chemotherapy.^108^ Reduced miR‐34b level was perceived as a predictor of unfavourable outcomes of OS patients and associated with MDR, which was reversed by administration of sirolimus in vitro.^116^ Besides, OS patients had a markedly higher level of serum miR‐21, which was associated with advanced Enneking stage and chemoresistance, and served as an independent prognostic factor for OS patients.^120^

Noticeably, some miRNAs have been reported to be rather promising therapeutic targets in preclinical or clinical studies in recent years (Table 3). The miR‐34 family including miR‐34a, miR‐34b, and miR‐34c, has been known as a tumour suppressor in cancers including OS and gained extensive attention.^121^, ^122^, ^123^, ^124^, ^125^, ^126^ In substantial preclinical studies, treatment with miR‐34 mimics was viewed as a novel miRNA‐target therapy in cancers.^121^, ^127^, ^128^ Besides, replenishment of miR‐34 encapsulated in lipid nanoparticles was demonstrated to exhibit an anticancer effect in several malignancies in a phase I clinical trail (NCT01829971).^129^ A recent study revealed that miR‐34 mimics could trigger the perturbation of microtubule network and cell death in OS cells, implicating its possibility as a therapeutic agent in OS.^130^ Recently, it is noted that miR‐34 mimic brought significant benefits for treatment of metastasis in OS mouse models.^131^ However, the optimal drug doses require further identification for application. The drug toxicity mentioned in this study was not associated with drug resistance. The loss of let‐7 is a prevalent phenomenon in various cancers, and its restoration obviously suppressed tumour growth and extended survival time in vivo.^132^ It is indicated that replenishing let‐7 might be a beneficial method in OS treatment, which remains to be investigated. MiR‐155 plays a critical and positive role in diverse cancer types.^133^ Recently, a study has reported a successful delivery of antimiR‐155 conjugated with a small peptide called pHLIP, and its therapeutic benefits without toxicity in a lymphoma mouse model,^134^ which requires verification in OS models. Increased level of miR‐221 which targets PTEN is of significance in hepatocellular carcinoma (HCC). A recent study revealed that antimiR‐221 modified with cholesterol significantly inhibited tumour growth and prolonged survival time in a HCC mouse model.^135^ However, the toxicity of cholesterol‐modified antimiR‐221 was not discussed in this study.

**Table 3:** Utility of OS‐related MiRNAs in cancer

microRNA	Study type	Cancer type	Treatment drug	Therapy effect	References
miR‐34a	Mouse model	Prostate cancer	Systemically delivered miR‐34a mimics	Inhibited prostate cancer metastasis and extended survival time	121
	Mouse model	Lung cancer	Systemically delivered miR‐34a mimics	A significant decrease in tumor burden	127
	Mouse model	Pancreatic cancer	A lipid‐based nanoparticle for systemic delivery with miR‐34a	Inhibited tumor growth	128
	Phase I clinical trial	Advanced solid tumors	A liposomal miR‐34a mimic, MRX34	Showed evidence of antitumor activity	129
	Mouse model	Osteosarcoma	Delivery of miR‐34a mimics	Suppressed pulmonary metastases and tumor progression, and improved the overall survival	131
miR‐155	Mouse model	Lymphoma	Delivery with antimiR‐155 conjugated with a small peptide	Showed evidence of antitumor activity	134
miR‐221	Mouse model	Hepatocellular carcinoma	Delivery with antimiR‐221 modified with cholesterol	Inhibited tumor growth and prolonged survival time	135

These miRNAs possess a unique advantage of clinical applications in OS treatment. For one thing, a single miRNA can simultaneously target multiple mRNAs implicated in one or several signal pathways. For another thing, OS has a complicated genetic background, and is characterized by inherent heterogeneity and aberrance of multiple genes.^3^, ^16^ Therefore, modulating the expression levels of these miRNAs might hold great promise for successful OS treatment and improving survival by effectively reversing chemoresistance. Some well‐studied miRNAs have been investigated in preclinical or clinical trials and exhibited their promising effects. Hence, it remains necessary to further confirm and test the clinical roles of these miRNAs.

## 6 CONCLUSIONS AND PERSPECTIVE

Drug resistance is the main reason for treatment refractoriness of OS. Recent studies have revealed the emerging roles of miRNAs in OS chemoresistance under the mechanisms of DDR, apoptosis avoidance, autophagy induction, activation of CSCs, and alteration in signal pathways. Perturbed DDR system, a hallmark of cancers, has been targeted to design effective DDR drugs for cancer therapy clinically.^136^ Autophagy can play both prosurvival and prodeath roles in cancer cells. Hence, researchers considered inhibiting its protective function and inducing cell death to treat cancers.^97^ The eradication of CSCs has been discussed for successful cancer treatment for years and exosomes were recently reported to have potential capability for targeting CSCs.^137^ These oncogenic or tumour suppressor miRNAs are expected to serve as promising candidates for prognosis and therapeutic targets, which provides a brand‐new outlook into better clinical treatment in OS. However, it still requires further identification, and more preclinical and clinical evidences in support of their future clinical applications.

Accumulated evidence has emphasized that besides miRNAs, other noncoding RNAs especially lncRNAs could function in OS drug resistance since lncRNAs account for a much bigger percentage than miRNAs in ncRNAs.^138^, ^139^ Some recent studies have reported the involvement of lncRNAs in OS chemosensitivity under the mechanisms of mediating MDR associated genes and signal pathways and interacting with miRNAs. LncRNA FOXC2 antisense RNA 1 (FOXC2‐AS1) contributed to poor response to DOX in OS patients by up‐regulating MDR associated proteins involving ABCB1and HIF1A.^140^ LncRNA OS doxorubicin‐resistance related up‐regulated lncRNA (ODRUL) was revealed to inhibit DOX sensitivity through inducing ABCB1 expression in OS cells.^141^ Overexpressed lncRNA HOXA transcript at the distal tip (HOTTIP) caused a resistance to CDDP by activating Wnt/β‐catenin signalling pathway.^142^ Of note, long intergenic noncoding RNA 161 (LINC00161) served as a tumour suppressor lncRNA with respect to OS resistance to CDDP by regulating the miR‐645‐IFIT2 signalling axis,^143^ which indicates an existence of a competitive endogenous RNA regulatory network. On one hand, a competition for the binding of miRNAs between lncRNAs and another nucleotide sequence or structure could have an effect on the translation of miRNAs’ targets.^144^ On the other hand, miRNAs might function as an upstream regulator of lncRNAs to regulate their expression levels.^145^ These interplays among endogenous RNAs are so complicated that demands for more potent evidence supports and deeper exploration of underlying molecular mechanisms. These results provided a new insight into identifying potential therapeutic targets for reversing OS chemoresistance based on the synergetic efforts of lncRNAs and miRNAs.

**Figure 4:**
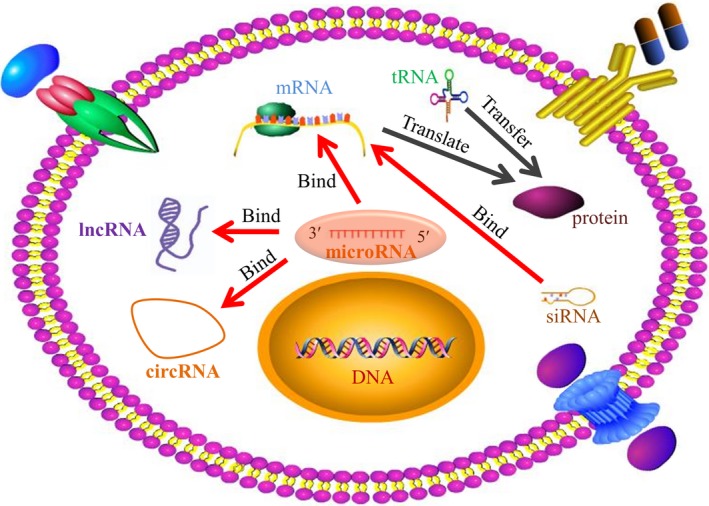
Competitive endogenous RNA regulatory network. Most of human genome DNA has transcripts. About 2% of transcripts code for protein synthesis. The remaining noncoding RNAs include miRNAs, lncRNAs, and cirRNAs. Numerous endogenous RNAs such as mRNAs, lncRNA, and cirRNAs are under the regulation of miRNAs, and they compete for the target binding of miRNAs

Of note, researchers have traditionally focused much on DNA, mRNA, and proteins, and viewed them as principal modulators and therapeutic targets. In recent two decades, accumulated evidence has revealed a competitive endogenous RNA regulatory network with the participation of miRNA, lncRNA, and circRNA (Figure 4). MiRNAs have gained increasing attention, and their antagonists or mimics have been designed in cancer therapy to reduce or elevate their previous levels, respectively.^146^, ^147^ On one hand, it's acknowledged that a single miRNA simultaneously targets several mRNAs implicated in several signal pathways, which brings great benefits to refractory cancers with genomic heterogeneity. On the other hand, a mRNA can be modulated by multiple miRNAs, implying a therapeutic strategy of applying different miRNA antagonists or mimics to effectively affect the specific target mRNA. However, there exist some disadvantages or challenges with respect to miRNA‐targeted strategy. At first, they may elicit broad effects and unexpected alterations of those unrelated genes targeted by same miRNAs, which obviously break the balance of gene expression profiles in cells. Besides, the existence of off‐target effect for miRNA antagonists cannot be ignored. Furthermore, the quick degradation and cellular delivery are two great challenges ought to be solved. Last but not least, it has been noticed that miRNAs can exert a different effect because of several influence factors such as agents, cell lines, cancer types, and so on. This implies careful and cautious choice of miRNA antagonists or mimics according to different conditions.

To sum up, this review focuses on drug resistance‐related miRNAs in OS through several molecular mechanisms, and provides insight in creating promising therapeutic strategies by targeting these miRNAs to reverse OS chemoresistance.

## CONFLICT OF INTEREST

The authors declare no conflict of interest.
